# Malignant solitary fibrous tumor of the pancreas: a case report

**DOI:** 10.1186/s40792-020-01067-6

**Published:** 2020-11-13

**Authors:** Yuka Taguchi, Takanobu Hara, Hiroaki Tamura, Masahito Ogiku, Mana Watahiki, Toru Takagi, Takashi Harada, Shinichiro Miyazaki, Tadataka Hayashi, Toshikazu Kanai, Hiroki Mori, Takachika Ozawa, Yoshiro Nishiwaki

**Affiliations:** 1 Department of Gastroenterological Surgery, Hamamatsu Medical Center, 328 Tomitsukacho, Naka-ku, Hamamatsu city, Shizuoka 432-8580 Japan; 2 Department of Surgery, Nagasaki University Graduate School of Biomedical Sciences, 1-7-1 Sakamoto, Nagasaki City, Nagasaki 852-8501 Japan; 3 Department of Pathology, Hamamatsu Medical Center, 328 Tomitsuka-cho, Naka-ku, Hamamatsu city, Shizuoka 432-8580 Japan

**Keywords:** Solitary fibrous tumor, Pancreas, Malignant, Surgery

## Abstract

**Background:**

Solitary fibrous tumors (SFTs) are rare tumors, mostly derived from connective tissue mesenchymal cells that arise from the pleura. There are very few reports of primary pancreatic SFT. Preoperative diagnosis is difficult owing to the lack of distinctive radiological findings. We report a case of pancreatic SFT with particularly rare malignant findings.

**Case presentation:**

A 60-year-old man was referred to the hospital because of a right upper quadrant mass and abnormal liver function test results. Contrast-enhanced computed tomography (CT) showed a well-defined enhanced tumor measuring approximately 8 cm in the pancreatic head. Magnetic resonance imaging (MRI) showed T1WI hypointensity, T2WI hyperintensity, and DWI hyperintensity. The main pancreatic duct and common bile duct were dilated owing to obstruction by the tumor. The following tumor markers were mildly elevated: carcinoembryonic antigen (CEA), carbohydrate antigen 19-9 (CA19-9), SPan-1, and DUPAN-2. The histological diagnosis obtained by endoscopic ultrasound-guided fine needle aspiration (EUS-FNA) was negative for pancreatic ductal carcinoma, malignant lymphoma and neuroendocrine tumor, suggesting the possibility of mesenchymal tumor, but the diagnosis was not confirmed. The patient was judged suitable for surgery and underwent subtotal stomach-preserving pancreatoduodenectomy with D2 lymph node dissection. On histopathological examination of the resected specimen, infiltrating spindle-shaped cells had proliferated, containing numerous mitotic figures, with necrotic findings inside the tumor. Immunostaining was positive for cluster of differentiation-34 (CD34), B cell CLL/lymphoma-2 (Bcl-2), and signal transducer and activator of transcription (STAT6). On the basis of these findings, a diagnosis of malignant pancreatic SFT was made. The patient remains free of recurrent disease after 12 months of follow-up without adjuvant therapy and he is being carefully followed up as an outpatient.

**Conclusions:**

We experienced a case of malignant pancreatic head SFT. Immunohistochemical staining of the extracted specimens was useful for diagnosis.

## Background

Solitary fibrous tumor (SFT) is a rare mesenchymal tumor typically located in the pleura that was first described in 1931 [1]. Several studies have since reported extra-pleural SFTs in almost every anatomic location. Most SFTs are characterized by a patternless distribution of both oval- and spindle-shaped cells in connective tissue. A correlation between either local recurrence or metastasis and histologic features such as necrosis, more than four mitoses per 10 high-power magnification fields (HPFs), increased nuclear pleomorphism, increased cellularity, tumor size larger than 10 cm, positive margins, and extra-thoracic location have been reported [2–4]. SFT of the pancreas was first reported in 1999 [5]. Because of its rarity, most reported pancreatic SFTs have demonstrated benign histopathologic features. We report a case of pancreatic SFT with malignant features confirmed by histopathology and immunohistochemical study.

## Case presentation

A 60-year-old male was referred to the hospital because of a right upper quadrant mass and abnormal liver function test results. He had no significant medical history.

Abnormal laboratory findings included elevated AST: 406 U/l, ALT: 397 U/l, total bilirubin: 1.01 mg/dl, direct bilirubin: 0.61 mg/dl, ALP: 4380 U/l, γ-GTP: 1548 U/l, and amylase: 509 U/l. The serum tumor markers carcinoembryonic antigen (CEA): 8.6 ng/ml, carbohydrate antigen 19-9 (CA19-9): 261 U/ml, SPan-1: 100 U/ml, and DUPAN-2: 750 U/ml were elevated, but soluble interleukin-2 receptor concentration was normal (310 U/ml).

**Fig. 1:**
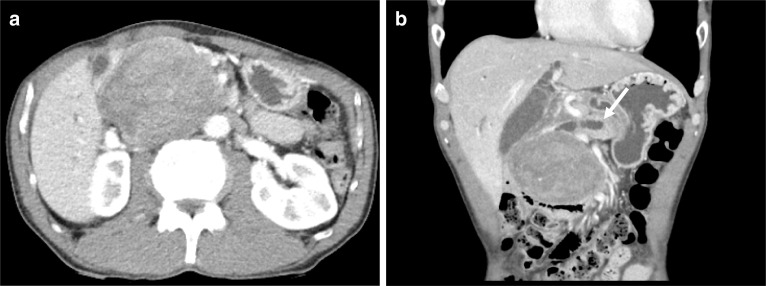
Abdominal CT. Enhanced CT shows a 7 × 9 × 7 cm tumor located in the pancreatic head (**a**). Dilatation of the distal pancreatic duct is seen (arrow) (**b**)

**Fig. 2:**
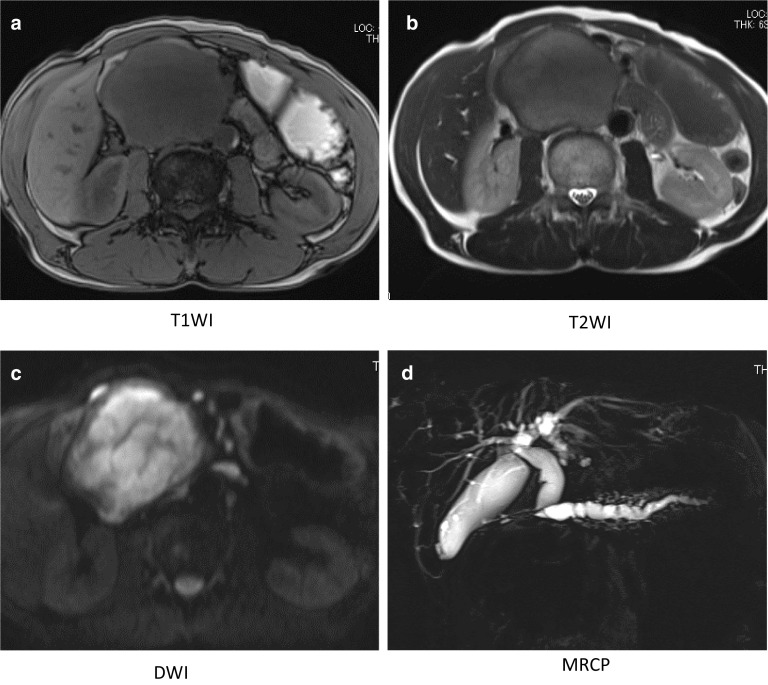
Abdominal MRI. MRI shows the tumor in the pancreatic head, with low signal intensity on T1-weighted imaging (**a**), low signal intensity on T2-weighted imaging (**b**), and high signal intensity on diffusion-weighted imaging (**c**). MR cholangio-pancreatography showing dilatation of intra- and extra-hepatic bile ducts and the pancreatic duct owing to tumor obstruction (**d**)

**Fig. 3:**
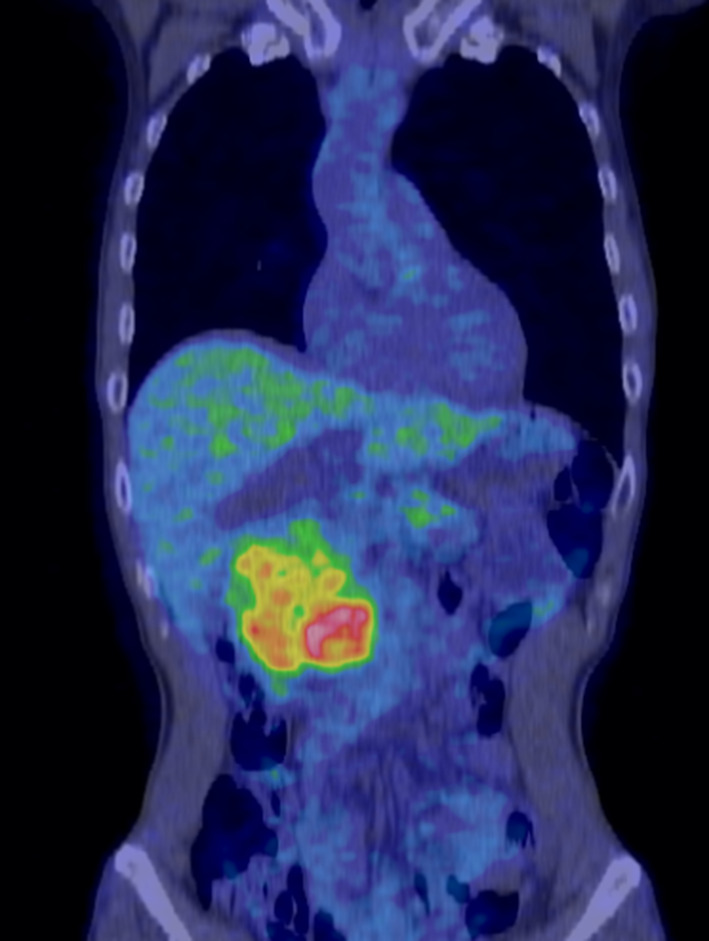
FDG-PET images. FDG-PET scan shows non-uniform increased uptake of fluorodeoxyglucose only in the pancreatic head (SUV max = 7.65)

Abdominal ultrasonography revealed a well-demarked heterogeneously solid mass with a diameter of 8 cm in the head of the pancreas. Contrast-enhanced computed tomography (CT) imaging of the abdomen confirmed a 7 × 9 × 7 cm diameter exophytic mass in the head of the pancreas. The tumor was hypodense in the arterial phase, and then became weakly but uniformly hyperdense in the delayed phase. There was biliary stricture, disruption of the main pancreatic duct, and obstructive pancreatitis, but no obvious infiltration of the surrounding organs or major blood vessels or enlarged lymph nodes (Fig. 1a, b). The mass was hypointense on T1-weighted magnetic resonance imaging (MRI), hyperintense on T2-weighted images, and diffusely hyperintense on diffusion-weighted images (DWI) (Fig. 2a–c). MR cholangio-pancreatography showed dilatation of intra- and extra-hepatic bile ducts and the pancreatic duct owing to tumor obstruction (Fig. 2d).[^18^F]-fluorodeoxyglucose positron emission tomography-computed tomography (FDG-PET) showed heterogeneous accumulation in the pancreatic head tumor (SUV max = 7.65). Abnormal accumulation suggesting distant metastasis or lymph node metastasis was not observed (Fig. 3). These findings were atypical for a pancreatic ductal carcinoma. Trans-duodenal tumor biopsy by endoscopic ultrasound-guided fine needle aspiration (EUS-FNA) was performed. Cytological examination showed a single pattern of atypical cells with increased chromatin granularity. Histopathological examination showed proliferating short spindle-shaped cells, relatively large N/C ratio, and scattered mitotic figures. Immunostaining was positive for cluster of differentiation (CD) 34 and CD56, partially positive for cytokeratin AE1/AE3, and negative for synaptophysin, chromogranin A, somatostatin receptor type 2 (SSTR2), CD117, DOG1, S-100, smooth muscle actin (SMA), desmin, and CD31.

**Fig. 4:**
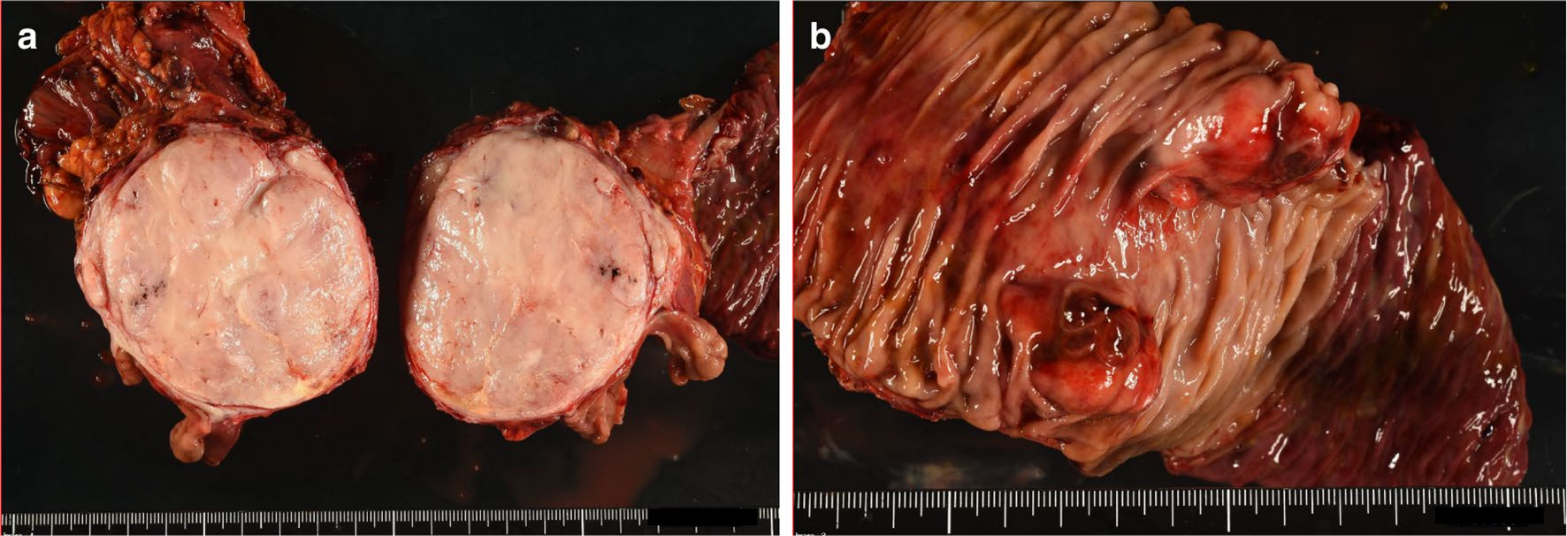
Macroscopic images of the resected specimen seen as a solid tumor located in the pancreatic head. The cut surface of the tumor is well-demarcated, heterogeneous, and yellowish-white in color (**a**). Small prominent lesions are seen in the duodenal mucosa, which were considered tumor invasion (**b**)

These findings suggested the possible diagnosis as SFT, extra-gastrointestinal stromal tumor (GIST), and neuroendocrine carcinoma, but the diagnosis was not confirmed because of limited amount of specimen and CD56 positive tumor. The EUS-FNA results indicated that the possibility of malignant lymphoma was low. Therefore, surgical resection was the therapy of choice for this large pancreatic head tumor. The patient underwent subtotal stomach-preserving pancreatoduodenectomy with D2 lymph node dissection. Intraoperative findings did not show ascites, peritoneal dissemination, or distant metastasis. A solid tumor measuring approximately 8 cm was found in the head of the pancreas, and the body of the pancreas showed obstructive pancreatitis. The tumor was firmly adherent to the surrounding tissues, such as the gallbladder, inferior vena cava, and mesentery owing to an inflammatory reaction. The inferior vena cava, superior mesenteric artery, and common hepatic artery were safely preserved by careful separation from the tumor. However, the border between the superior mesenteric vein and the tumor was partly unclear, and partial resection was required. The resected tumor was a yellowish-white, well-circumscribed mass measuring 8 × 8 × 6 cm and was located in the pancreatic head (Fig. 4a). A prominent lesion was observed in the duodenum, which was considered to be tumor invasion (Fig. 4b).

**Fig. 5:**
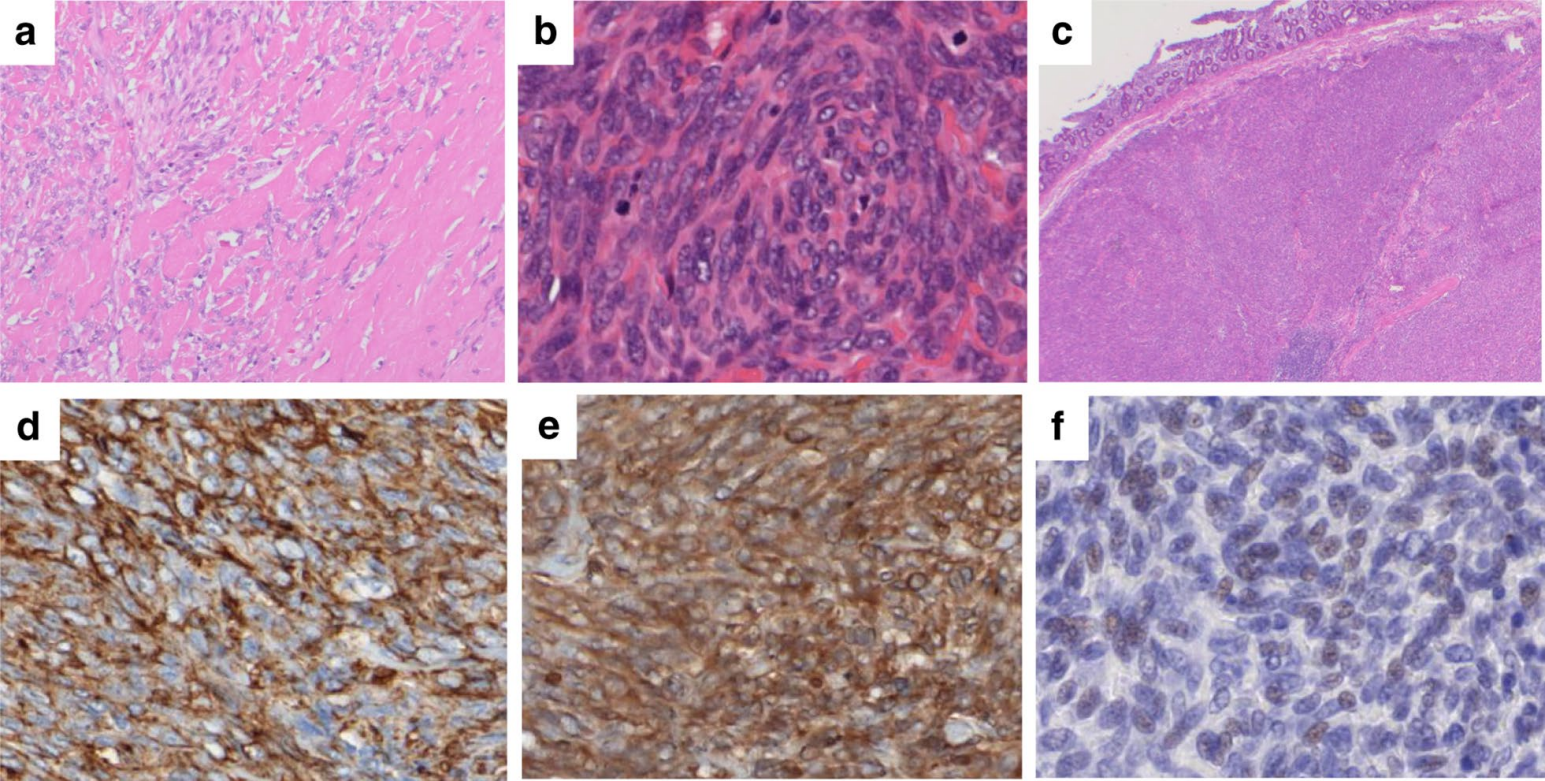
Histopathological findings of the resected specimen. Invasive growth of proliferated spindle-shaped cells in the pancreatic tumor. Hyalinized fibrosis is present at the periphery of the tumor (× 100) (**a**). The tumor showed high cellularity, increased mitotic figures (12/10 HPFs), and nuclear pleomorphism (increased N/C ratio) (× 200) (**b**). Tumor infiltration was observed on the duodenal side in contact with the main lesion (**c**). Immunohistochemically, the tumor cells were positive for CD34 (× 200) (**d**) and Bcl-2 (× 200) (**e**), and weakly positive for STAT6 (× 200) (**f**)

Pathological examination of the resected specimen demonstrated proliferating spindle-shaped cells involving normal pancreatic tissue, and it was considered that most of the cells were excreting and some were invasive (Fig. 5a). Fibrosis accompanied by hyalinization was observed at the margin of the tumor, and there were necrotic foci inside the tumor. Twelve mitotic figures were observed in 10 HPFs (Fig. 5b). In addition, venous infiltration and duodenal infiltration (Fig. 5c) were observed on the duodenal side in contact with the main lesion, suggesting a highly malignant tumor.

Immunohistochemical analysis of the resected tumor revealed that the tumor cells expressed positive results for CD34 (Fig. 5d), vimentin, and Bcl-2 (Fig. 5e), focally positive for cytokeratin AE1/AE3, and weakly positive for STAT6 (Fig. 5f). The tumor cells were negative for CD117, DOG1, SMA, desmin, S-100, synaptophysin, and chromogranin A. On the basis of the histology and immunostaining profile, the tumor was diagnosed as a malignant SFT of the pancreas.

The patient’s postoperative course was uneventful except for gastric stasis, which was treated with conservative management. The patient was discharged 22 days after surgery, and he remains free of recurrent disease after 12 months of follow-up without adjuvant therapy.

## Discussion

SFT was reported by Klemperer et al. in 1931 as a tumor of the pleura [1]. SFT is a rare tumor, and Gold et al. reported that it constituted less than 2% of all soft tissue tumors [2]. SFT is a mesenchymal tumor typically located in the thoracic cavity, but the tumor can also be found in soft tissues and organs throughout the body [6]. SFT of the pancreas is extremely rare, with a total of 29 reported cases, including the present case [5, 7–33]. A summary of these cases is shown in Tables 1 and 2. The median age at diagnosis is 53 years, and there is no gender difference (14 males and 15 females reported). The most common tumor site is the pancreatic head, with 17 cases; 9 cases were reported in the pancreatic body and 3 cases in the pancreatic tail. Pancreatic resection was performed in 28 patients, and pancreatoduodenectomy and distal pancreatectomy were often performed, as with other pancreatic malignancies. Furthermore, mass enucleation was selected in 5 cases and central pancreatic resection in 1 case.

**Table 1:** Patient characteristics of pancreatic solitary fibrous tumors

Author	Year	Age, sex	Chief complaints	Size (cm)	Location	Primary diagnosis	Treatment
Lüttges et al. [5]	1999	50, F	Incidental	5.5	Body	NET	DP
Chatti et al. [7]	2006	41, M	Abdominal pain	13	Body	NET	Enucleation
Gardini et al. [8]	2007	62, F	Abdominal pain	3	Head	NET	PD
Miyamoto et al. [9]	2007	41, F	Abdominal pain	2	Head–body	NET	Enucleation
Srinivasan et al. [10]	2008	78, F	Back pain weight loss	5	Body	Mesenchymal tumor	DP
Kwon et al. [11]	2008	54, M	Incidental	4.5	Body	NET, SPT	Median segmentectomy
Ishiwatari et al. [12]	2009	58, F	Incidental	3	Head	NET	PD
Chetty et al. [13]	2009	67, F	Incidental	2.6	Head	NET	PD
Sugawara et al. [14]	2010	55, F	Incidental	7	Head	NA	PD
Santos et al. [15]	2012	40, M	Incidental	3	Body	NA	Partial pancreatectomy
Tasdemir et al. [16]	2012	24, F	Epigastric pain	18.5	Head	Mesenchymal tumor	Enucleation
Azadi et al. [17]	2012	57, M	Incidental	3.1	Tail	NA	DP
van der Vorst et al. [18]	2012	67, F	Abdominal pain	2.8	Head	NET	Enucleation
Yamanashi et al. [19]	2012	50, M	Incidental	10	Tail	NEC	DP
Chen et al. [20]	2013	49, F	Abdominal pain	13	Head		PD
Hwang et al. [21]	2014	53, F	Incidental	5.2	Head	NET, SPT	PD
Han et al. [22]	2015	77, F	Jaundice	1.5	Head	SFT	Conservative
Estrella et al. [23]	2015	52, F	Jaundice	15	Head	NET	PD
Baxter et al. [24]	2015	58, F	Abdominal pain	3.5	Head	NET, GIST, SPT, SFT	PD
Paramythiotis et al. [25]	2016	55, M	Abdominal pain	3.6	Body	NET, SPT, GIST	DP
Murakami et al. [26]	2016	82, M	Hypokalemia hypertension, edema	6	Tail	NET	DP
Spasevska et al. [27]	2016	47, M	Epigastric pain jaundice	3.5	Head	Cystadenocarcinoma	PD
Clare et al. [28]	2017	39, F	Incidental	2.2	Head	NA	PD
Sheng et al. [29]	2017	1, M	Jaundice	2	Head	NA	PD
D'Amico et al. [30]	2017	52, M	Incidental	2	Body	NET	Enucleation
Oana et al. [31]	2017	73, M	Abdominal discomfort	7.5	Head	NET, ACC, GIST	Partial pancreatectomy
Geng et al. [32]	2020	48, M	Hypoglycemia	6.5	Body	SFT, liver metastasis	TACE, DP left lateral liver sectionectomy
Qian et al. [33]	2020	46, M	Hypoglycemia	7	Body	NEC, liver metastasis	TACE, DP left lateral liver sectionectomy
Present case		60, M	Palpable mass	8	Head	NEC, GIST, SFT	PD

*NET* neuroendocrine tumor, *DP* distal pancreatectomy, *PD* pancreatoduodenectomy, *SPT* solid pseudopapillary tumor, *NA* not applicable, *NEC* neuroendocrine carcinoma, *SFT* solitary fibrous tumor, *GIST* gastrointestinal stromal tumor, *ACC* acinar cell carcinoma, *TACE* transarterial chemoembolization

**Table 2:** Histological features and outcomes of pancreatic solitary fibrous tumors

Author	Positive immunohistochemistry	Malignant features	Diagnosis of malignant SFT	Recurrence	Outcome	Follow-up
Lüttges et al. [5]	CD34, CD99, Bcl-2, vimentin	No	No	No	Alive	20 mo
Chatti et al. [7]	CD34, CD99, Bcl-2, vimentin	No	No	No	Died postoperative complications	3 d
Gardini et al. [8]	CD34, CD99, Bcl-2, vimentin, SMA (focal)	NA	No	No	Alive	16 mo
Miyamoto et al. [9]	CD34, Bcl-2	No	No	No	Alive	7 mo
Srinivasan et al. [10]	CD34, Bcl-2	No	No	No	Alive	7 mo
Kwon et al. [11]	CD34, CD99, vimentin	No	No	No	NA	NA
Ishiwatari et al. [12]	CD34, Bcl-2	Necrosis	No	No	Alive	42 mo
Chetty et al. [13]	CD34, CD99, Bcl-2	No	No	No	Alive	6 mo
Sugawara et al. [14]	CD34	No	No	No	NA	NA
Santos et al. [15]	CD34, beta-catenin	No	No	No	NA	NA
Tasdemir et al. [16]	CD34, Bcl-2, beta-catenin, vimentin, Ki67 < 2%	No	No	No	Alive	3 mo
Azadi et al. [17]	CD34, Bcl-2, Ki67 < 5%	No	No	No	NA	NA
van der Vorst et al. [18]	CD34, CD99, Bcl-2	No	No	No	NA	NA
Yamanashi et al. [19]	CD34, vimentin, Bcl-2	Intra-pancreatic metastasis, necrosis, > 2 mitoses/HPFs, hypercellularity	Yes	Intra-pancreatic	Alive	32 mo
Chen et al. [20]	CD34, Bcl-2, vimentin, CD68, muscle-specific actin	Necrosis	No	No	Alive	30 mo
Hwang et al. [21]	CD34, Bcl-2, muscle-specific actin, CD10, ER, PR	No	No	No	Alive	30 mo
Han et al. [22]	CD34, CD99	No	No	–	No progression	10 mo
Estrella et al. [23]	CD34, Bcl-2, keratin (rare), p16, p53	Nuclear atypia, necrosis 17 mitoses/10 HPFs,	Yes	No	Alive	40 mo
Baxter et al. [24]	CD34, Bcl-2	NA	No	No	NA	NA
Paramythiotis et al. [25]	CD34, CD99, Bcl-2, vimentin, S-100 (focal)	No	No	No	Alive	40 mo
Murakami et al. [26]	STAT6, CD34, Bcl-2, ACTH (focal), POMC (focal), NSE (focal)	No	No	No	Died sepsis	4 mo
Spasevska et al. [27]	CD34, vimentin, CD99, Bcl-2 (focal), nuclear beta-catenin (focal)	No	No	No	Died postoperative complications	1 wk
Clare et al. [28]	STAT6, CD34, Bcl-2, CD56, cytokeratin CAM5.2, AE1/AE3	6/10 HPFs	Yes	No	Alive	40 mo
Sheng et al. [29]	CD34, vimentin, SMA (focal), Ki67 < 3%	Mild–moderate nuclear pleomorphism 2–5/10 HPFs hypercellularity	No	No	Alive	12 mo
D'Amico et al. [30]	STAT6, CD34	No	No	No	Alive	24 mo
Oana et al. [31]	CD34, Bcl-2	No	No	No	Alive	36 mo
Geng et al. [32]	STAT6, CD34, Bcl-2, CD31, PHH-3, D2-40, Ki67 > 10%	4–5/10 HPFs necrosis	Yes	Residual liver tumor ( +)	Alive	6 mo
Qian et al. [33]	STAT6, CD34, Bcl-2, Ki67 10%	Heterotypic cell 4–5/10 HPFs local infarction	Yes	NA multiple recurrence	Alive	10 mo
Present case	STAT6, CD34, Bcl-2, vimentin, cytokeratin AE1/AE3(focal)	Hypercellularity 12/10 HPFs necrosis invasive growth	Yes	No	Alive	12 mo

*SFT* solitary fibrous tumor, *HPFs* high-power fields, *CD* cluster of differentiation, *Bcl-2* B cell CLL/lymphoma-2, *STAT6* signal transducer and activator of transcription 6, *ER* estrogen receptor, *PR* progesterone receptor, *SMA* smooth muscle actin, *NA* not applicable

Pancreatic SFT shows a well-defined mass with an internal heterogeneous contrast effect with CT, and exhibits hypointensity on T1WI and hyperintensity on T2WI with MRI, in most cases. These features are atypical, which makes it difficult to distinguish SFT from other soft tissue tumors [17, 32]. In our case, the tumor was visualized as a well-defined tumor with a weak contrast effect, and exhibited hypointensity on T1WI and hyperintensity on T2WI, with diffuse strong hyperintensity on DWI. On the basis of the above findings, we considered that the differential diagnoses of the tumor should include malignant lymphoma, neuroendocrine tumor, acinar cell carcinoma, extra-GIST, and SFT, but it was not possible to make a diagnosis, preoperatively. Histopathological examination, including immunohistochemical staining, was considered important, and a tumor biopsy was performed. However, biopsy also could not confirm the diagnosis, but malignant lymphoma could be ruled out, so we selected surgery as treatment.

Histologically, SFT shows two features: a patternless appearance in which elliptical- to spindle-shaped tumor cells grow randomly, and a hemangiopericytic growth pattern owing to vascular proliferation and perivascular sclerosis. Because SFT is a mesenchymal tumor, immunohistochemical staining is positive for CD34 and vimentin, and negative for mesothelial cell-derived cytokeratin and epithelial membrane antigen. Staining is also negative for S-100, which is positive for neurogenic tumors, and negative for c-kit, which is positive for GIST. These features are useful for distinguishing SFT from other mesenchymal tumors [6, 19]. Recently, it was revealed that NAB2–STAT6 fusion was the driver mutation in SFT, and the transcriptional repressor of the cell division pathway is converted to the transcriptional activator [34]. Therefore, STAT6 has been proven to be more sensitive (98%) and specific (85%) for SFT [32]. The present case was finally diagnosed as a SFT of the pancreas because the tumor was positive for STAT6.

Most SFTs are benign, but some are known to recur or metastasize. Previous reports have shown the histopathologic features of malignant SFT as (1) high cellularity; (2) more than 4 mitotic figures per 10 HPFs; (3) nuclear pleomorphism; (4) hemorrhage and necrosis; (5) tumor diameter ≥ 10 cm, and (6) positive margins [2–4]. Demicco et al. also reported that age > 55 years is a poor prognostic factor [35]. In the present case, the tumor was positive for STAT6, also showed high cellularity, increased mitotic figures (12/10 HPFs), nuclear pleomorphism (increased N/C ratio), necrosis inside the tumor, and invasive proliferative findings. The patient was 60 years old, so all findings fulfilled the malignant features except for tumor size. On the basis of the histology and immunohistochemical staining profile, we made a diagnosis of malignant SFT.

Unlike patients in other reports, our case was characterized by partially positive expression of cytokeratin AE1/AE3. SFT is classically negative for cytokeratin, but Cavazza et al. reported a malignant pleural SFT in which the majority of the neoplastic cells strongly expressed cytokeratin AE1/AE3. The authors reported that cytokeratin AE1/AE3-positive cells were lightly scattered in the pleural SFT primary lesion, and that 70% of the tumor cells were positive in the intrathoracic disseminated lesion 4 years after resection [36]. According to previous reports of pancreatic SFT, two cases were positive for cytokeratin and keratin that also had histopathologically malignant findings with numerous mitotic figures [23, 28]. Including our case, in pancreatic SFT, like pleural SFT, cytokeratin positivity may indicate high malignant potential.

To our knowledge, only five cases of pancreatic SFT had malignant findings, and two had distant metastases at the time of diagnosis. Twenty-two patients who underwent surgical treatment and had no malignant findings were free from recurrence; their prognosis was considered favorable. In our case, no recurrence was observed 12 months postoperatively. Because a case of recurrence and metastasized pancreatic malignant SFT has been reported, periodic follow-up with image examination is recommended. To date, there is no established postoperative adjuvant therapy or treatment for recurrence. We await the future accumulation of cases.

## Conclusion

We experienced a case of pancreatic head SFT. Immunohistochemical staining of the excised specimen was useful for diagnosis. Careful follow-up is demanding because of several malignant features.

## Data Availability

The authors declare that all the data in this article are available within the article.

## Abbreviations

Bcl-2: B cell CLL/lymphoma-2
CA19-9: Carbohydrate antigen 19-9
CD: Cluster of differentiation
CEA: Carcinoembryonic antigen
CT: Computed tomography
DWI: Diffusion-weighted image
EUS-FNA: Endoscopic ultrasound-guided fine needle aspiration
FDG-PET: [^18^F]-fluorodeoxyglucose positron emission tomography-computed tomography
GIST: Gastrointestinal stromal tumor
HPFs: High-power magnification fields
MRI: Magnetic resonance imaging
SFT: Solitary fibrous tumor
SMA: Smooth muscle actin
SSTR2: Somatostatin receptor type 2
STAT6: Signal transducer and activator of transcription
